# Single-Band NDVI Spatial Downscaling: Synthetic Benchmarking and Landsat-8/Sentinel-2 Cross-Sensor Evaluation

**DOI:** 10.3390/s26206601

**Published:** 2026-10-18

**Authors:** Sihan Yu, Jinhua Li, Xiaoxiang Yuan, Yin Wang

**Affiliations:** 1 Earthquake Agency of Ningxia Hui Autonomous Region, Yinchuan 750001, China; 2 Institute of Earthquake Forecasting, China Earthquake Administration, Key Laboratory of Earthquake Forecasting and Risk Assessment, Ministry of Emergency Management, CEA Key Laboratory of Earthquake Prediction, Beijing 100036, China; lijinhua26@mails.ucas.ac.cn; 3 Institute of Engineering Mechanics, China Earthquake Administration, Key Laboratory of Earthquake Disaster Prevention and Mitigation, Ministry of Emergency Management, Key Laboratory of Earthquake Engineering and Engineering Vibration, China Earthquake Administration, Harbin 150080, China

**Keywords:** NDVI, spatial downscaling, image super-resolution, Sentinel-2, Landsat-8, cross-sensor transfer, synthetic degradation, EDSR

## Abstract

Medium-resolution NDVI products can smooth small vegetation patches, land-cover boundaries, and local gradients. This study presents a two-part evaluation of single-band NDVI spatial downscaling: a controlled synthetic Sentinel-2 benchmark and a separate transfer assessment on real Landsat-8/Sentinel-2 imagery. The contribution is the evaluation design and evidence; we assess, but do not propose, the published EDSR architecture. We compared EDSR with bicubic interpolation, SRCNN, and VDSR. Training used 2200 precomputed Sentinel-2 NDVI scenes; validation used 330 geographically separate Anhui patches; and testing used 300 Zhengzhou patches. Synthetic LR inputs were generated from NDVI using a fixed 3× bicubic operator. The cross-sensor set comprised 389 co-located image pairs acquired on 15 and 17 May 2023, with 117 pairs used for shared linear calibration and 272 held out. EDSR achieved 35.70 dB PSNR and 0.920 SSIM on the synthetic test set. On held-out cross-sensor pairs, its mean PSNR was 25.58 dB before and 26.40 dB after calibration, gains of 0.61 and 0.62 dB over bicubic, respectively. After calibration, the paired EDSR–VDSR PSNR difference was 0.232 dB (95% bootstrap CI, 0.203–0.263). These findings describe performance under the two evaluated protocols; the cross-sensor results apply to the available paired acquisitions.

## 1. Introduction

Vegetation is a fundamental component of terrestrial ecosystems and is central to agricultural production and environmental monitoring. Its spatial distribution and growth status are closely related to carbon cycling and regional climate. The normalized difference vegetation index (NDVI) characterizes vegetation cover and condition using the contrast between red and near-infrared reflectance [1]. Because it is straightforward to calculate and physically interpretable, NDVI is widely used for vegetation-index performance assessment [2], crop-condition monitoring [3], and land-cover analysis [4].

Spatial resolution describes the ground area represented by each image pixel. For the same geographic area, coarser imagery contains fewer pixels, and each pixel is more likely to mix multiple cover types. In heterogeneous landscapes, medium-resolution NDVI can therefore combine vegetation and non-vegetation signals. This can smooth NDVI patterns in fragmented farmland, peri-urban zones, and narrow strips of vegetation, obscuring local texture and boundaries. Improving spatial detail in NDVI can support fine-scale applications such as precision agriculture and urban-green-space monitoring. Early NDVI downscaling methods often used statistical regression with high-resolution auxiliary data. Gu and Wylie [5] used multi-date Landsat imagery and data-mining methods to downscale 250-m MODIS growing-season NDVI to 30 m. Although interpretable, these approaches depend on auxiliary imagery acquired at similar times and on assumptions linking predictors to NDVI, which can limit their transferability. Spatiotemporal fusion methods followed. Gao et al. [6] introduced STARFM, which combines the temporal frequency of MODIS with the spatial detail of Landsat. Hwang et al. [7] extended this approach to track vegetation dynamics over complex terrain. These methods combine temporal and spatial information, but their performance can be affected by image quality, cloud contamination, scale mismatch, and mixed pixels. They may also recover fine spatial detail only partially.

Deep-learning methods for image super-resolution learn nonlinear mappings from low-resolution (LR) to high-resolution (HR) images. SRCNN introduced a CNN-based approach to single-image super-resolution [8]. VDSR used a deeper network with residual learning to address vanishing gradients [9]. EDSR removed batch-normalization layers, refined the residual architecture, and applied residual scaling [10]. Later models extended this approach with channel attention (RCAN) [11] and window-based Transformers (SwinIR) [12]. In remote sensing, deep learning has been used to downscale MODIS NDVI with SAR data [13], enhance Sentinel-2 multispectral imagery [14], and improve the spatial detail of Landsat archives for long-term vegetation monitoring [15]. Supervised training requires co-registered LR–HR pairs with a known scale relationship, which are uncommon in real acquisitions. Researchers therefore often create synthetic pairs by applying a specified degradation operator, enabling controlled comparisons. Evaluation on real cross-sensor observations complements these tests by assessing transfer across sensors and acquisition conditions [16].

Recent remote-sensing studies have combined sensor-aware degradation with newer network architectures. Qiu et al. [17] used edge-guided attention with a sensor-aware degradation model for cross-sensor super-resolution. Sambandham et al. [18] jointly addressed spectral and spatial differences between Landsat-8 and Sentinel-2 and evaluated downstream vegetation-index applications. Aybar et al. [19] compared CNN, Mamba, and Swin Transformer models in a Sentinel-2 super-resolution framework with radiometric and spatial consistency constraints. These studies inform the methodological context of our work. Their reported scores are not directly comparable with our single-band NDVI benchmark because the studies use different sensors, spectral bands, spatial scales, and reference protocols.

The contribution of this study is a two-part evaluation that connects a reproducible synthetic Sentinel-2 benchmark with a separate held-out Landsat-8/Sentinel-2 transfer assessment for single-band 3× NDVI downscaling. We compare bicubic interpolation with three established CNN pipelines and evaluate the published EDSR architecture without proposing architectural modifications. We report results on geographically independent validation and test sets, cross-sensor metrics before and after shared calibration, and uncertainty for paired performance differences.

## 2. Materials and Methods

### 2.1. Study Area and Data

The training data comprised 2200 Sentinel-2 NDVI scenes from the publicly available Dataset of Sentinel-1 SAR and Sentinel-2 NDVI Imagery [20]. The scenes were collected from geographically diverse regions worldwide between January 2017 and March 2024, within the latitude range of 40° S to 40° N, and were supplied as precomputed NDVI rasters. These NDVI scenes were used as the high-resolution source for constructing the synthetic training pairs described in Section 2.3.

The validation set comprised 330 patches from Anhui Province, and the test set comprised 300 patches from Zhengzhou, Henan Province. Both areas were geographically separate from the training data; the Anhui patches were used for validation and model selection, and the Zhengzhou patches were reserved for independent testing.

The Anhui validation area includes forests, croplands, water bodies, and built-up land. The Zhengzhou test area includes a mixture of urban, agricultural, vegetated, and water-covered landscapes.

We also compiled a cross-sensor dataset over Zhengzhou from Landsat-8 30-m NDVI acquired on 15 May 2023 and Sentinel-2 10-m NDVI acquired on 17 May 2023. Vegetation conditions may have changed during the two-day interval, and the acquisitions also differ in sensor response and atmospheric processing. We randomly divided the 389 co-located pairs at the pair level, assigning 117 pairs (30%) to calibration and 272 pairs (70%) to independent evaluation.

For this cross-sensor dataset, we calculated NDVI from red and near-infrared surface reflectance using the following equation:

(1) $$NDVI=\frac{\left(NIR-Red\right)}{\left(NIR+Red\right)}$$

We used Sentinel-2 bands B4 and B8 and Landsat-8 OLI bands 4 and 5. We converted digital numbers (DNs) to surface reflectance using each product’s scale and offset metadata. For Landsat-8 Collection 2 Level-2 surface reflectance, we applied the scale factor and offset as follows [21]:

(2) $$\rho_{SR}\;=\;DN\;\times \;0.0000275\;-\;0.2$$

For Sentinel-2 Level-2A, BOA reflectance was calculated using the band-specific offset and quantification value reported in the product metadata [22]:

(3) $$\rho_{BOA,i}\;=\;\frac{{DN}_{i}\;+\;O_{i}}{Q_{BOA}}$$

Here, *O_i_* denotes the band-specific BOA_ADD_OFFSET, and Q_BOA denotes the BOA_QUANTIFICATION_VALUE. In the May 2023 Sentinel-2 granule, Q_BOA was 10,000, and the offsets for B04 and B08 were both −1000 DN. These reflectance conversions apply only to the cross-sensor dataset. By contrast, the training archive consisted of precomputed Sentinel-2 NDVI rasters, which we used as supplied [20].

We used the 2200 precomputed 10-m Sentinel-2 NDVI scenes from the public dataset [20] as supplied and generated their corresponding 30-m LR images using bicubic resampling. For validation and testing, we cropped low-cloud Sentinel-2 Level-2A scenes over southern Anhui Province and Zhengzhou, Henan Province, into non-overlapping patches. We discarded patches containing invalid values, cloud, or cloud shadow. This yielded 330 Anhui validation patches and 300 independent Zhengzhou test patches. Neither evaluation set shares imagery with the training data. We generated LR counterparts using the same bicubic operator. Table 1 lists the scene identifiers and acquisition dates.

For cross-sensor evaluation, we used Landsat-8 OLI Level-2 imagery over Zhengzhou, acquired two days before the Sentinel-2 scene, to calculate 30-m NDVI. The same spatial crops from the Landsat and Sentinel-2 images yielded 389 co-located pairs, with Landsat 30-m NDVI as input and Sentinel-2 10-m NDVI as the reference.

To align the cross-sensor inputs and reference, we used the Landsat raster geotransform to define the 30-m grid and derived a 10-m target grid at three times the pixel density over the same footprint. The Sentinel-2 NDVI raster was reprojected and cubic-resampled to this target grid. We then extracted each LR tile (170 × 170 pixels) and its HR counterpart (510 × 510 pixels) from the same aligned window. Both source rasters use EPSG:32649. This procedure aligns the pixel grids using product georeferencing; no additional image-based subpixel registration or independent residual-displacement accuracy assessment was performed.

### 2.2. Methodology

**Figure 1:**
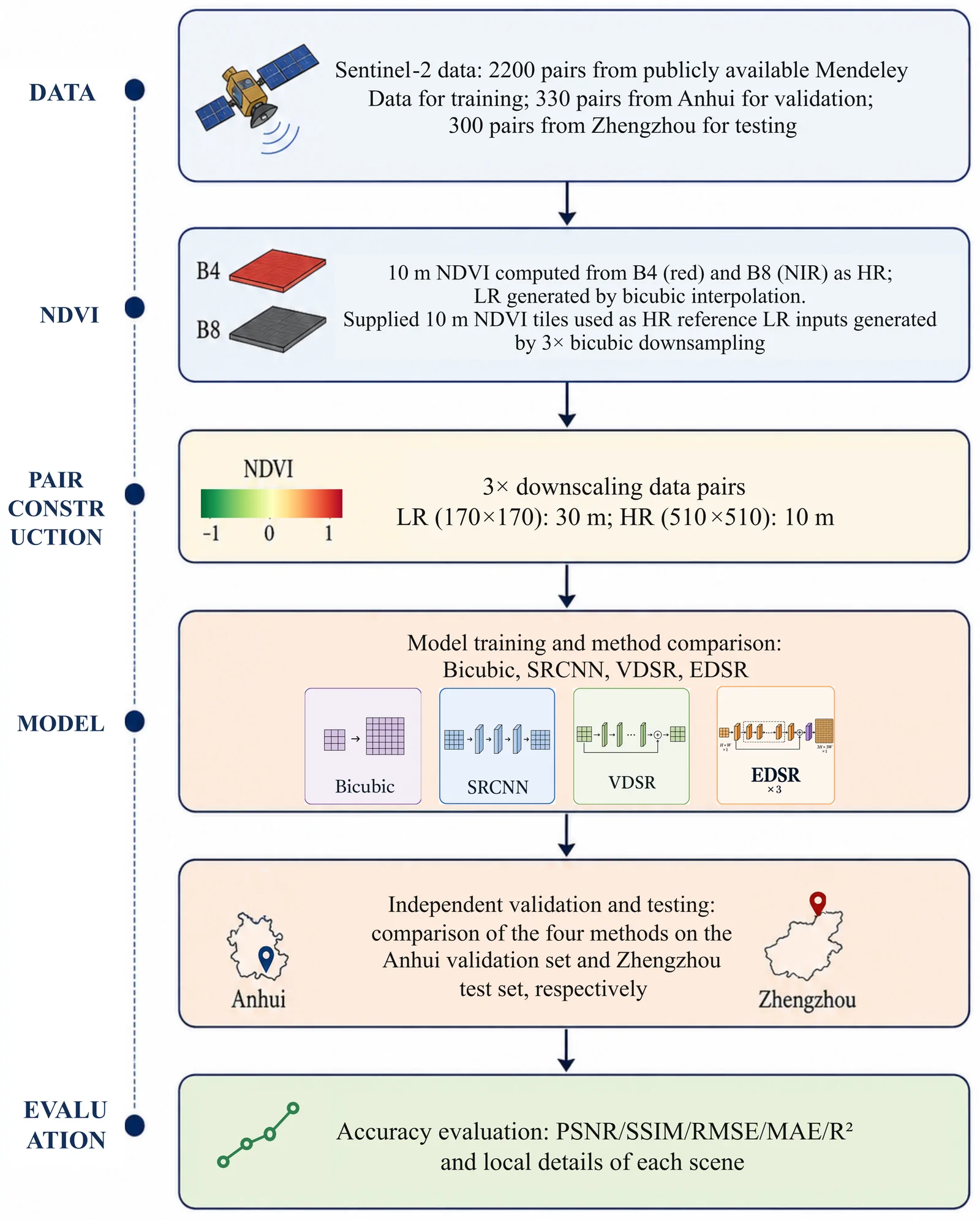
Workflow for NDVI spatial downscaling.

Figure 1 summarizes the two evaluation workflows. In the synthetic Sentinel-2 branch, we used the supplied NDVI scenes to construct 3× LR–HR pairs for training and evaluated the models on independent Sentinel-2 validation and test datasets. We compared EDSR, SRCNN, and VDSR with bicubic interpolation under the same protocol. In the separate cross-sensor branch, we evaluated transfer on the held-out Landsat-8/Sentinel-2 pairs and applied a shared calibration fitted on the separate calibration subset where indicated. We report quantitative metrics and visual analyses for the relevant datasets.

### 2.3. Construction of LR–HR Pairs

The synthetic task uses the supplied 10-m NDVI product as the HR target. We downsampled it to 30 m using bicubic resampling (scale factor 3) to create the LR input. This product-level design gives all methods the same deterministic LR–HR pairs and evaluates reconstruction of the supplied NDVI product. Previous studies have documented how NDVI varies with spatial scale over heterogeneous surfaces [23] and compared two processing orders: aggregating fine-resolution NDVI versus calculating NDVI from aggregated red and near-infrared reflectance [24]. These operations can produce different values, with the magnitude depending on scene composition and spatial heterogeneity [23,24]. Previous studies have also demonstrated NDVI product downscaling with higher-resolution auxiliary data [13,25,26]. Our audit confirmed that all 2200 archived LR training tiles match bicubic resampling of their paired HR tiles. Together, these pairs define a reproducible synthetic 30-to-10 m benchmark. The degradation operator is bicubic resampling of NDVI, not an explicit model of sensor optics or point-spread response. For the bicubic baseline, we upsampled each 30-m LR image to 10 m using bicubic interpolation.

For locally processed Sentinel-2 Level-2A scenes used for validation, testing, and the Sentinel-2 cross-sensor reference, we assigned NoData to pixels that fell outside SCL classes 4–7, had a non-positive sum of red and near-infrared reflectance, or had non-finite reflectance or NDVI. For Landsat-8 cross-sensor NDVI, we applied the Collection 2 QA_PIXEL mask and excluded non-finite reflectance or NDVI values. These reflectance- and mask-based procedures apply to scenes generated from source bands; we did not apply them to the 2200 training tiles because those were supplied as precomputed NDVI and used as provided. During evaluation-patch extraction, we excluded any candidate patch containing invalid values, NoData, cloud, or cloud shadow. This complete-valid-support rule keeps masked pixels out of the evaluation metrics. We retained valid negative NDVI values.

For model training and inference, valid NDVI values were clipped to [−1, 1] and linearly normalized to [0, 1] using the following:

(4) $${NDVI}_{norm}=\frac{\mathrm{clip}\left(NDVI,-1,1\right)+1}{2}$$

After inference, we clipped inverse-normalized predictions to [−1, 1] before saving the learned-model GeoTIFFs. When red and near-infrared reflectances are non-negative, NDVI is bounded by this interval [1]. Clipping therefore maps estimates outside the valid range to the nearest endpoint rather than discarding the pixel. The network output layer is unconstrained, so predictions may fall outside this range before clipping. We did not archive the pre-clipping outputs; their frequency therefore cannot be quantified from the saved results. All learned-model predictions reported here are the clipped outputs.

(5) $$\hat{NDVI}=2{\hat{NDVI}}_{norm}-1$$

### 2.4. Downscaling Models

Four methods were compared. Bicubic interpolation served as the non-learned baseline. SRCNN [8] uses a shallow three-layer network to learn the LR-to-HR mapping directly. VDSR [9] uses a deeper network to predict the residual between an interpolated input and the reference. EDSR [10] was the primary model.

EDSR follows the published architecture; its implementation settings are summarized in Table 2.

### 2.5. Training Configuration

We adapted the published reference implementations of SRCNN, VDSR, and EDSR to single-band NDVI pairs. We trained all three models on the same workstation using Adam, a learning rate of 1 × 10^−4^, and a maximum of 100 epochs with early stopping. The checkpoints selected for inference came from epochs 55, 55, and 86 for SRCNN, VDSR, and EDSR, respectively. Each LR/HR tile measured 170 × 170/510 × 510 pixels. Training crops measured 96 × 96 LR pixels for SRCNN and 48 × 48 LR pixels for VDSR and EDSR; the corresponding HR crops were three times larger. SRCNN and VDSR used MSE as the training objective, whereas EDSR used L1, consistent with the canonical objectives described in the original papers [8,9,10]. For SRCNN, MSE updated the weights. We computed validation-set L1 at each epoch only to select and save the checkpoint with the lowest validation L1 for inference; it did not update the weights. The detailed training parameters are presented in Table 3.

### 2.6. Cross-Sensor Evaluation Protocol

Landsat-8 OLI and Sentinel-2 MSI differ in spectral response, band width, spatial sampling, viewing geometry, and acquisition time; these factors can affect their NDVI values [27]. The cross-sensor experiment therefore evaluates the complete input-to-reference pipeline for the paired acquisitions rather than isolating sensor response.

The geospatial grid alignment described in Section 2.1 establishes a common map-coordinate sampling grid but does not quantify residual image-to-image displacement. We did not perform an additional image-based subpixel registration or calculate a residual-shift statistic; possible remaining geolocation mismatch is therefore part of the cross-sensor evaluation setting.

We report metrics before and after calibration. The uncorrected metrics compare each method’s output directly with the Sentinel-2 reference and therefore include both reconstruction error and the original inter-sensor discrepancy. The bias-corrected metrics apply a shared global linear adjustment, fitted on a separate calibration subset, to adjust for a first-order systematic difference:

(6) $$\hat{y}corr\;=\;a\;\cdot \;\hat{y}\;+\;b$$

where ŷ is the downscaled Landsat NDVI, and a and b are scalar correction coefficients.

To prevent information leakage, we randomly split the 389 co-located Landsat-8/Sentinel-2 pairs at the pair level: 117 pairs (30%) for calibration and 272 pairs (70%) for held-out evaluation. We used only the calibration subset to estimate the global linear correction; the evaluation subset was not used to fit its coefficients. For each calibration pair, we averaged corresponding 3 × 3 blocks of 10-m Sentinel-2 NDVI pixels to obtain a 30-m reference. We then regressed these values against the corresponding 30-m Landsat-8 NDVI using ordinary least squares on valid co-located pixels. The resulting shared linear correction was defined as follows:

(7) $$\hat{y}corr\;=\;1.18877\;\hat{y}\;-\;0.11530$$

Let x denote Landsat-8 NDVI at 30 m and y the corresponding Sentinel-2 NDVI averaged to 30 m for calibration. Using the 117 calibration pairs, which contained 3,376,512 valid co-located pixels, we fitted the shared relation y = 1.18877x − 0.11530 (in-sample R^2^ = 0.9174, RMSE = 0.06934, and MAE = 0.05378). We applied the same coefficients, without method-specific refitting, to the Bicubic, SRCNN, VDSR, and EDSR reconstructions in the separate 272-pair held-out subset. We then evaluated each reconstruction against the aligned 10-m Sentinel-2 reference. This calibration captures a shared first-order relationship in the paired acquisitions. Because the images were acquired two days apart and differ in sensor and processing characteristics, we do not interpret it as a sensor-only correction.

### 2.7. Accuracy Metrics

We assessed reconstruction quality using peak signal-to-noise ratio (PSNR) [28], structural similarity (SSIM) [29], mean absolute error (MAE) [30], root mean square error (RMSE) [31], and the coefficient of determination (R^2^).

(8) $$PSNR=10\log_{10}\left(\frac{MAX^{2}}{MSE}\right)$$

We first excluded non-finite and NoData pixels in either the reference or prediction, then clipped the remaining NDVI values to [−1, 1] before calculating the metrics. We set MAX = 2 in the PSNR equation to represent the full NDVI range.

(9) $$SSIM=\frac{\left(2\mu ₓ\mu ᵧ+C_{1}\right)\left(2\sigma ₓᵧ+C_{2}\right)}{\left[\left(\mu {ₓ}^{2}+\mu {ᵧ}^{2}+C_{1}\right)\left(\sigma {ₓ}^{2}+\sigma {ᵧ}^{2}+C_{2}\right)\right]}$$

Here, μ, σ, and σ__xy_ denote local means, standard deviations, and covariance, respectively; C_1_ and C_2_ are stabilizing constants. SSIM ranges from 0 to 1, with values closer to 1 indicating greater structural similarity. MAE and RMSE quantify differences between a reconstruction and its reference. MAE gives equal weight to all absolute errors and is less sensitive to large errors than RMSE:

(10) $$MAE=\left(\frac{1}{N}\right)\Sigma \left|y_{i}-{\hat{y}}_{i}\right|$$

(11) $$RMSE=\sqrt{\left[\left(\frac{1}{N}\right)\Sigma {\left(y_{i}-{\hat{y}}_{i}\right)}^{2}\right]}$$

(12) $$R^{2}=1-\frac{\Sigma {\left(y_{i}-{\hat{y}}_{i}\right)}^{2}}{\Sigma {\left(y_{i}-\bar{y}\right)}^{2}}$$

We calculated image-reconstruction metrics for each patch or image pair and averaged them over the relevant set. For the 300-patch Zhengzhou synthetic test set, we estimated 95% percentile-bootstrap confidence intervals for paired PSNR differences by resampling patch IDs 20,000 times (NumPy default_rng seed = 42). For cross-sensor EDSR–VDSR comparisons, we resampled the 272 held-out image pairs as paired units for 20,000 replicates (seed = 42). The intervals for bias-corrected predictions are conditional on the shared calibration coefficients fitted to the separate 117-pair calibration set and do not include uncertainty in those coefficients. Learned-model inference scripts clipped predictions to [−1, 1] before saving. Bicubic interpolation produced 51 values above +1 and none below −1; we clipped these values before calculating the metrics. We calculated Sobel-gradient-magnitude MAE across the 300 patches using the saved, range-constrained predictions.

## 3. Results

### 3.1. Performance on Independent Sentinel-2 Data

Table 4 and Table 5 report mean metrics for the Anhui validation set and Zhengzhou test set. The ranking was consistent across all five metrics in both sets: EDSR > VDSR > SRCNN > bicubic. On validation, EDSR exceeded bicubic by 2.08 dB in PSNR and 0.041 in SSIM; on test, the gains were 2.03 dB and 0.043, respectively. On the geographically separate Zhengzhou test set, which shared no imagery with the training or validation sets, PSNR differed from the validation result by no more than 0.22 dB. Based on unrounded per-patch means, the absolute validation–test differences were 0.00413–0.00572 for SSIM, 0.00081–0.00094 for MAE, 0.00065–0.00074 for RMSE, and 0.00554–0.00816 for R^2^. These are descriptive cross-region similarities, not a formal equivalence test or proof that overfitting is absent. The paired bootstrap estimated EDSR’s PSNR advantage at 2.034 dB (95% CI, 1.968–2.102) over bicubic, 1.403 dB (1.339–1.467) over SRCNN, and 0.284 dB (0.272–0.296) over VDSR; all three confidence intervals excluded zero. EDSR ranked first on all five metrics in each of the 300 patches. Because all patches came from one Zhengzhou scene, these intervals describe within-scene patch variability and do not establish geographic replication.

The EDSR–VDSR gap was 0.29 dB on validation and 0.28 dB on test. These differences were substantially smaller than the PSNR gains of VDSR and EDSR over bicubic on test (1.75 and 2.03 dB, respectively). The main improvement was therefore the gain of the learned methods over interpolation; differences among the learned models were narrower.

To isolate the effect of NDVI calculation order while holding the spatial filter constant, we used the 17 May 2023 Sentinel-2 scene to compare two procedures based on a 3 × 3 arithmetic mean. In the first, we calculated NDVI at 10 m and then averaged it. In the second, we first averaged BOA-corrected red and near-infrared reflectance and then calculated NDVI. Among 13,093,595 common valid 30-m pixels, the band-first minus NDVI-first difference had a mean of −0.00131, an MAE of 0.00459, an RMSE of 0.01054, and a 95th-percentile absolute difference of 0.01789. Differences exceeded 0.01 for 12.49% of pixels and 0.05 for 0.57%. This single-scene analysis quantifies sensitivity to calculation order under a shared mean filter. It does not compare the bicubic training operator with a sensor point-spread function or measure the effect of retraining a model with band-first data.

The 0.28-dB EDSR–VDSR test-set advantage should be considered alongside model size. EDSR has 20.369 million parameters, compared with 0.666 million for VDSR, or about 30.6 times as many. We did not log inference latency and therefore make no speed comparison.

### 3.2. Visual Comparison

**Figure 2:**
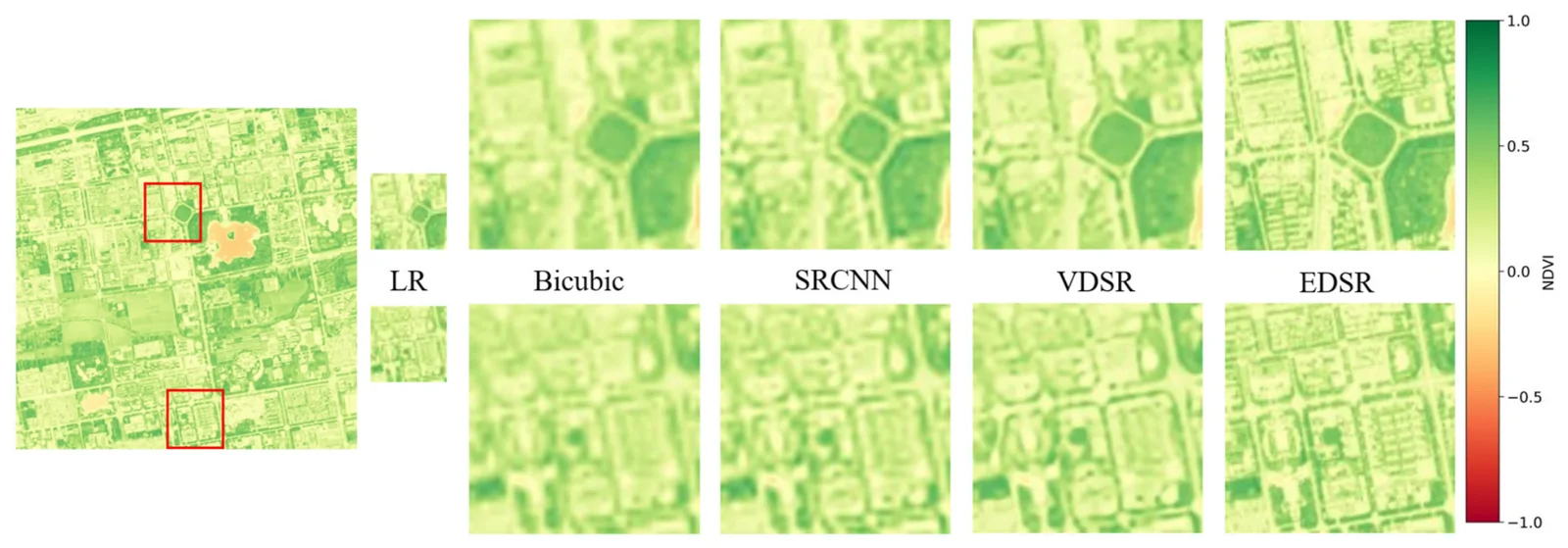
Visual comparison of validation scene 1 (bicubic, SRCNN, VDSR, EDSR).

**Figure 3:**
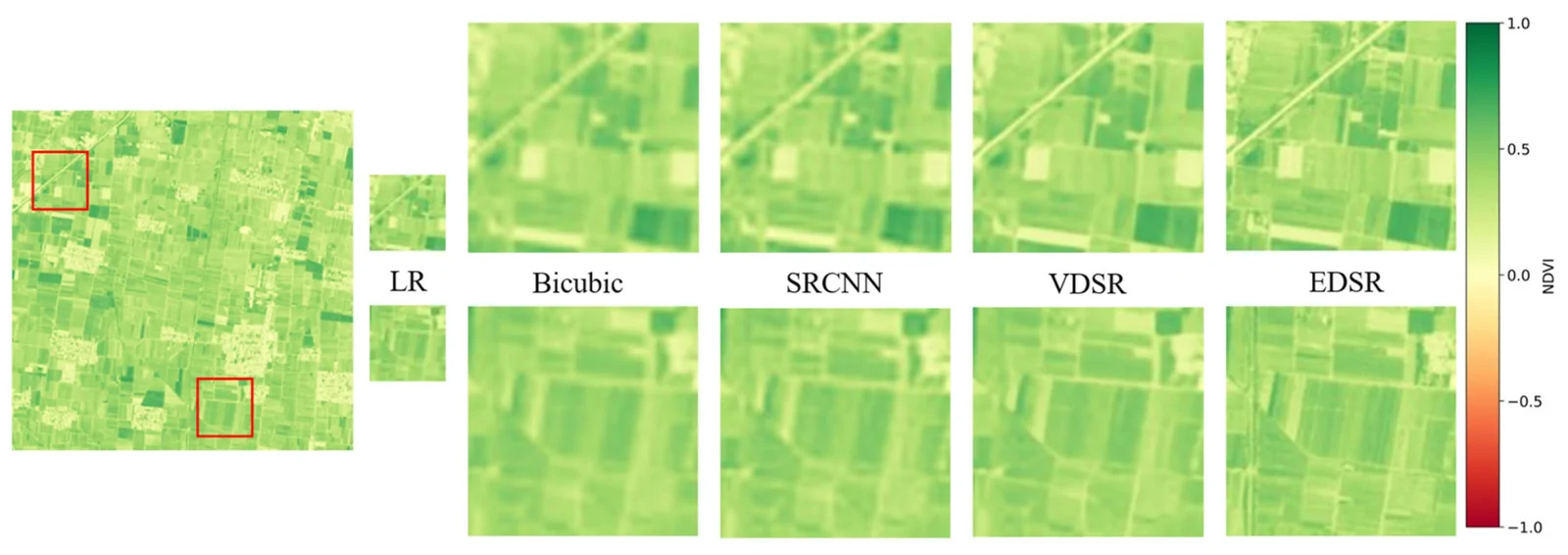
Visual comparison of validation scene 2 (bicubic, SRCNN, VDSR, EDSR).

**Figure 4:**
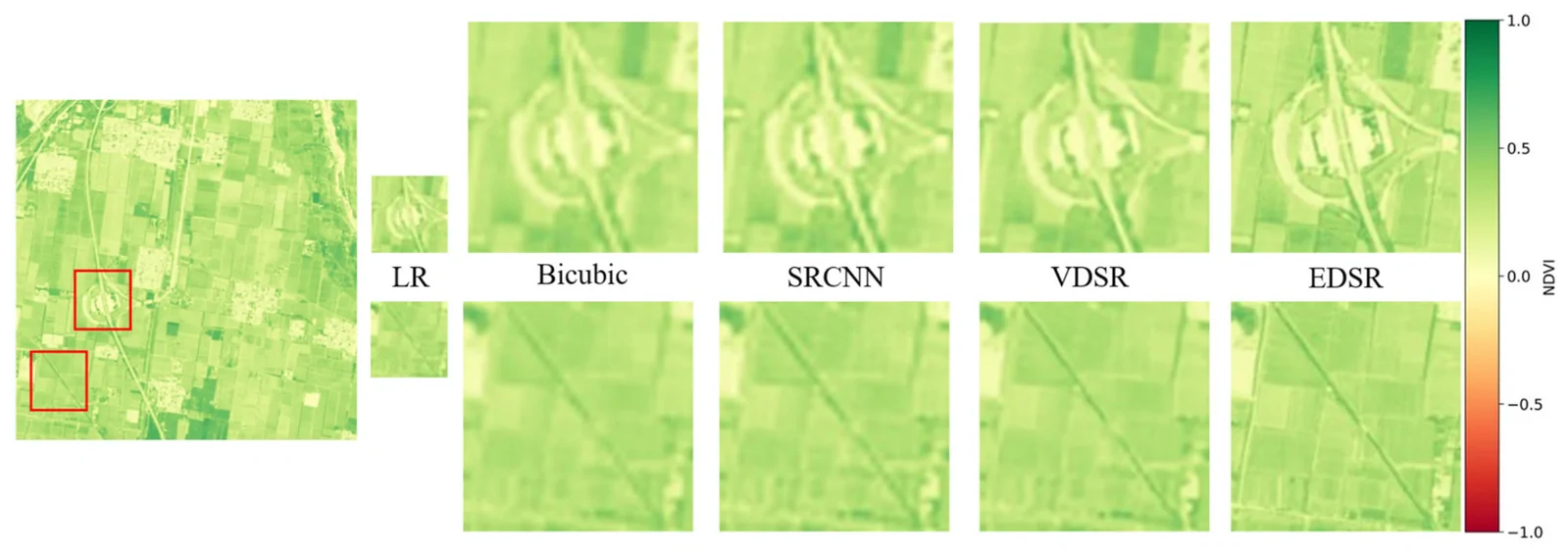
Visual comparison of test scene 1 (bicubic, SRCNN, VDSR, EDSR).

**Figure 5:**
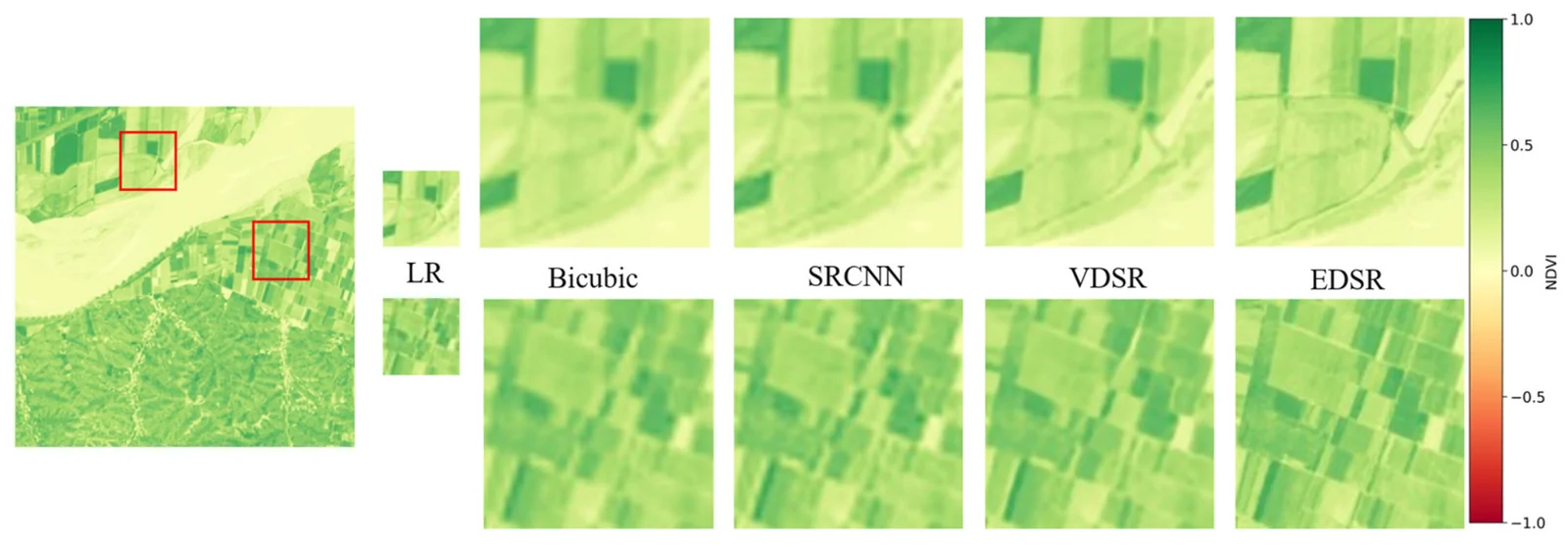
Visual comparison of test scene 2 (bicubic, SRCNN, VDSR, EDSR).

Bicubic reconstructions showed visible smoothing near vegetation boundaries and in small fragmented patches. SRCNN produced sharper edges than bicubic interpolation but remained blurred in fine structures and areas with strong local gradients. VDSR and EDSR recovered textures closer to the reference. Among the methods, EDSR retained the most detail at patch boundaries and object outlines and showed the least visible distortion, consistent with the quantitative ranking. Figure 2 and Figure 3 show the validation examples; Figure 4 and Figure 5 show the test examples.

Across the 300-patch Zhengzhou test set, Sobel-gradient-magnitude MAE was 0.01387 for EDSR, 0.01468 for VDSR, 0.01691 for SRCNN, and 0.01902 for bicubic interpolation. This metric is the mean absolute difference between each reconstruction’s Sobel gradient magnitude and that of the HR reference; lower values indicate closer agreement in local gradient magnitude. EDSR had the lowest value among the methods. We calculated this metric from the saved predictions after clipping them to [−1, 1].

### 3.3. Cross-Sensor Evaluation on Landsat-8/Sentinel-2 NDVI

We evaluated the trained models on the cross-sensor dataset, using Landsat-8 OLI 30-m NDVI as input and Sentinel-2 MSI 10-m NDVI as the reference. Following the calibration and held-out evaluation split described in Section 2.6, Table 6 and Table 7 report results for the 272 held-out pairs.

**Figure 6:**
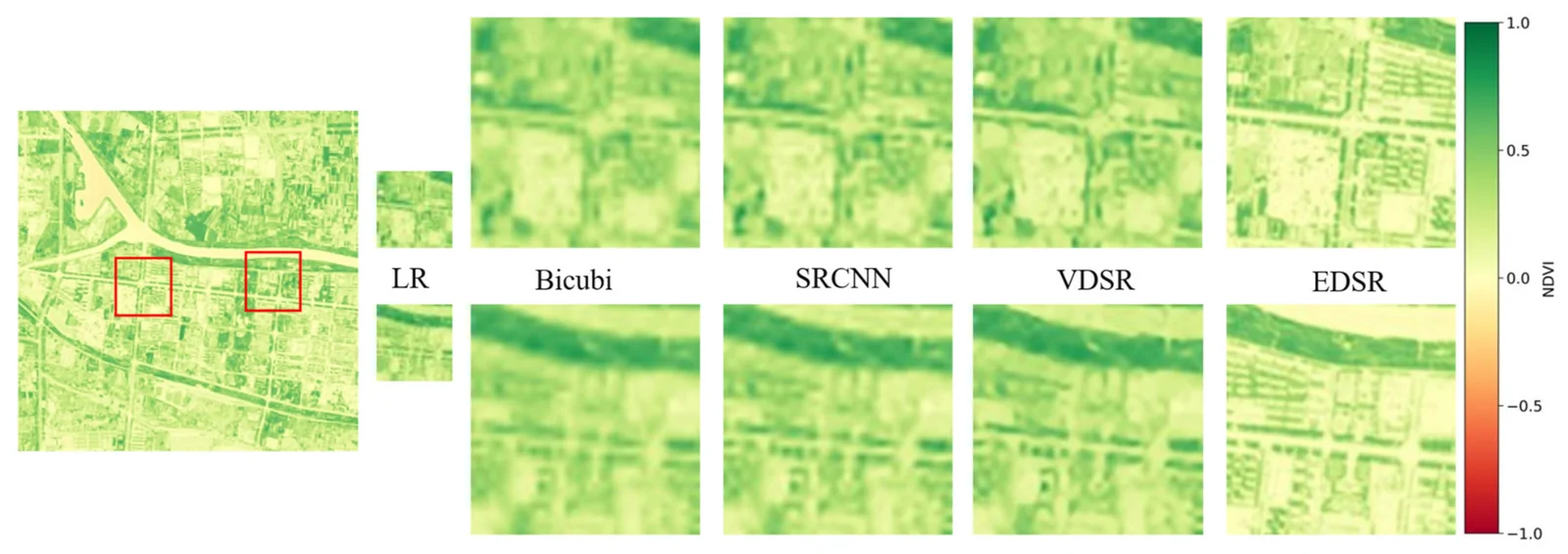
Cross-sensor visual comparison of tile 0104 (LR, bicubic, SRCNN, VDSR, and EDSR).

**Figure 7:**
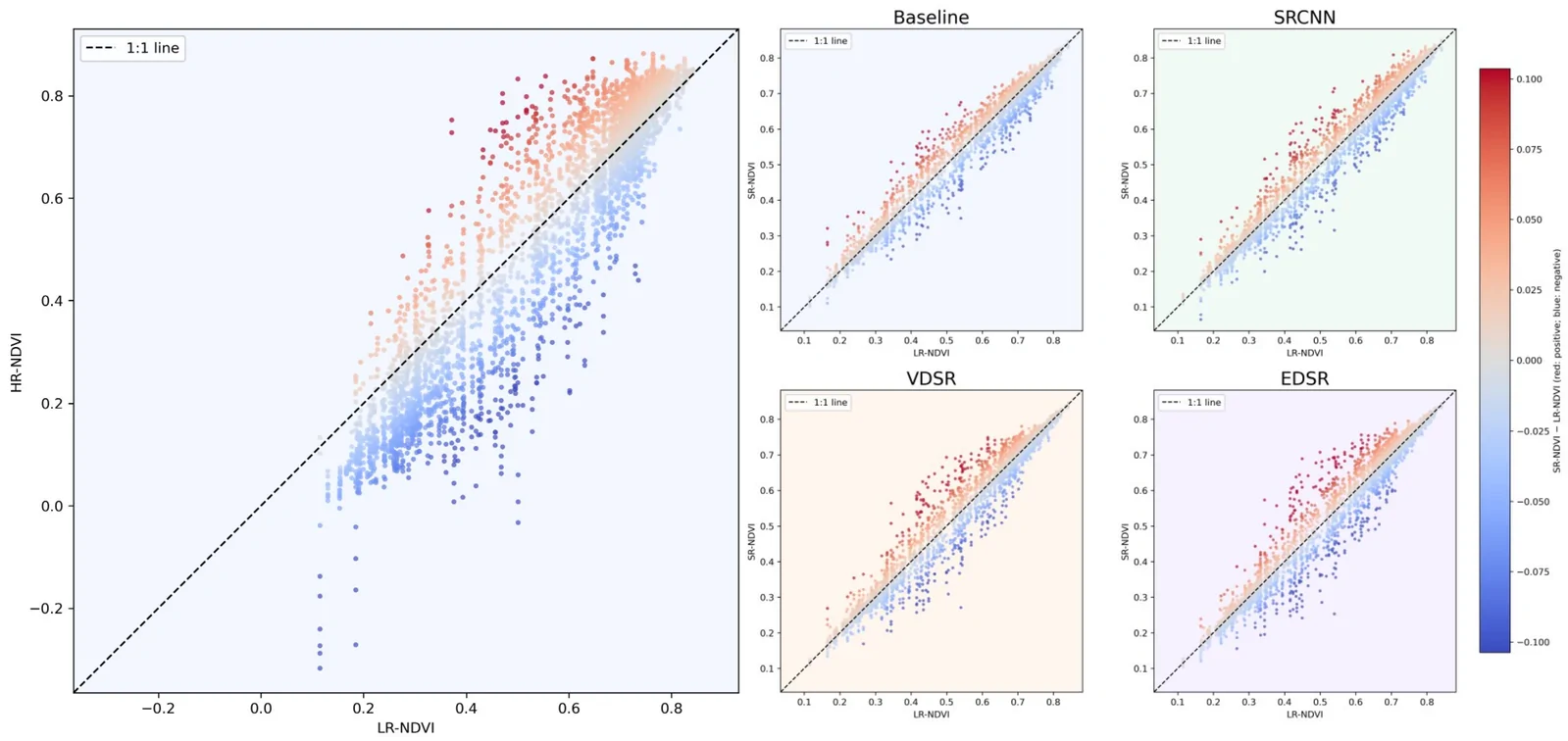
Pixel-wise LR–HR and LR–SR NDVI relationships for cross-sensor tile 0104.

**Figure 8:**
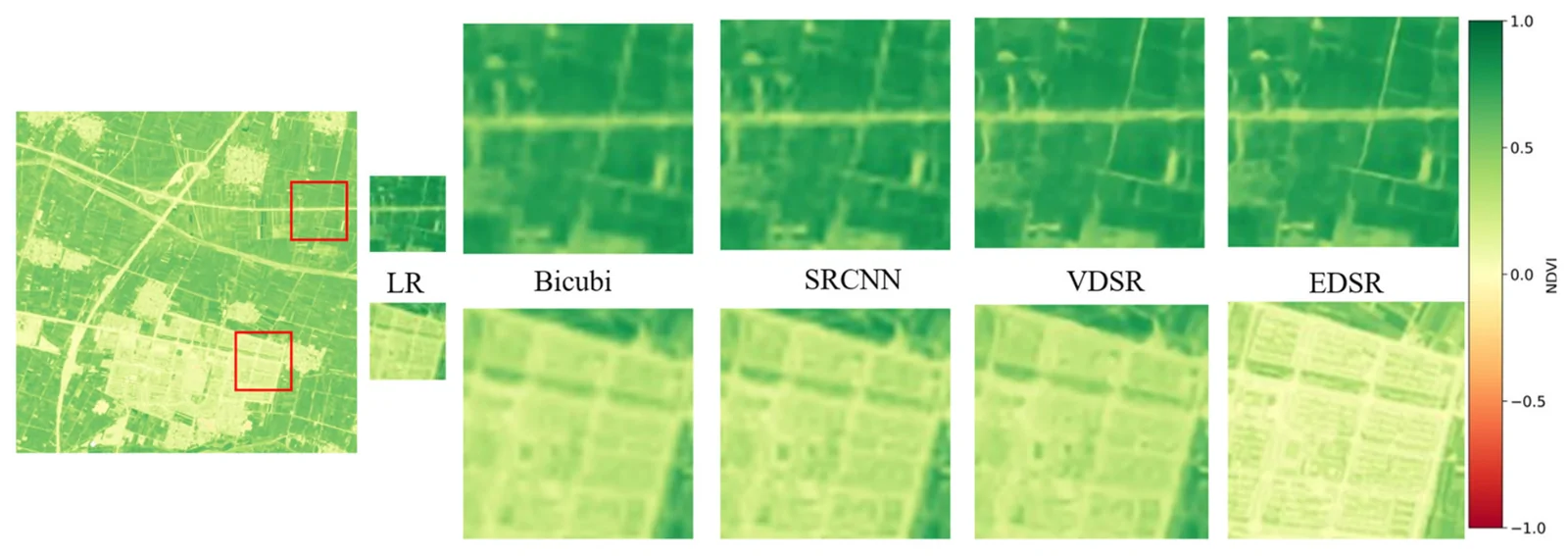
Cross-sensor visual comparison of tile 0224 (LR, bicubic, SRCNN, VDSR, and EDSR).

**Figure 9:**
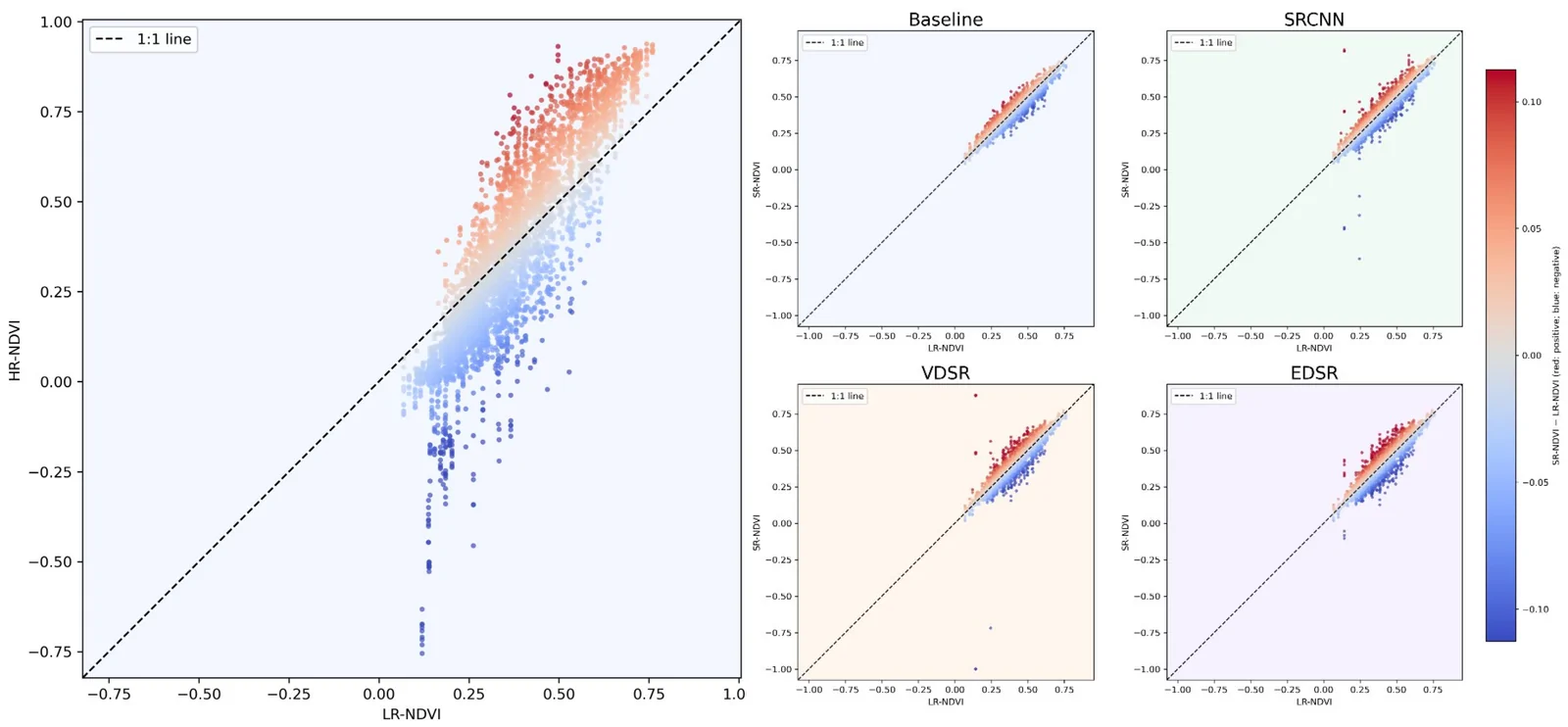
Pixel-wise LR–HR and LR–SR NDVI relationships for cross-sensor tile 0224.

Table 6 and Table 7 report the means of the pair-specific metrics over all 272 held-out image pairs. For EDSR, shared calibration changed mean PSNR from 25.58 to 26.40 dB, SSIM from 0.7279 to 0.7703, RMSE from 0.1073 to 0.0979, MAE from 0.0828 to 0.0723, and R^2^ from 0.7764 to 0.8102. EDSR’s mean PSNR gain over bicubic was 0.61 dB before calibration and 0.62 dB afterward, compared with 2.03 dB on the synthetic test set. The paired mean EDSR–VDSR PSNR difference was 0.263 dB (95% pair-bootstrap CI, 0.239–0.288) before calibration and 0.232 dB (0.203–0.263) after calibration; both intervals excluded zero. The bias-corrected interval is conditional on the fitted shared calibration coefficients. EDSR had the highest mean scores among the four methods after calibration. For tile 0104, Figure 6 shows the image patch and Figure 7 shows its pixel-wise LR–HR/LR–SR NDVI relationships; Figure 8 and Figure 9 present the same two displays for tile 0224. These examples illustrate local pixel-value relationships, while Table 6 and Table 7 summarize all 272 held-out pairs.

## 4. Discussion

### 4.1. Architecture Ranking Is Stable, but the Margins Are Narrow

EDSR ranked first among the three tested CNN pipelines on both synthetic Sentinel-2 sets and the held-out cross-sensor subset. Its PSNR advantage over VDSR was 0.28 dB on the synthetic test set and 0.23 dB after cross-sensor calibration. Across the 272 held-out pairs, the paired mean EDSR–VDSR difference after calibration was 0.232 dB (95% CI, 0.203–0.263), conditional on the fixed calibration coefficients. Because the models used different training objectives (MSE for SRCNN and VDSR; L1 for EDSR) and we did not conduct a controlled loss ablation, this ranking compares complete training pipelines and cannot isolate architecture effects.

### 4.2. Synthetic and Cross-Sensor Evaluations Give Different Gains

EDSR’s PSNR gain over bicubic was 2.03 dB under the matched synthetic protocol and 0.61 dB before calibration and 0.62 dB after calibration on the held-out cross-sensor pairs. These evaluations address complementary questions: reconstruction under a controlled NDVI degradation and transfer between the available Landsat-8/Sentinel-2 acquisitions. The cross-sensor result reflects the sensors’ spectral and spatial characteristics, acquisition timing, processing, and geospatial alignment; we therefore interpret it as performance for this specific paired acquisition.

EDSR retained a positive PSNR gain over bicubic interpolation on the held-out cross-sensor pairs, indicating an advantage under this pairing. Future evaluation with temporally matched observations and sensor-aware degradation can test whether this gain generalizes across acquisition conditions.

### 4.3. Limitations

This study establishes a reproducible single-band NDVI benchmark, and its findings apply to the settings evaluated here. The synthetic LR data were generated by applying one fixed bicubic operator to NDVI; this operator does not model optical blur, the sensor point-spread function, or sensor noise. Our calculation-order sensitivity analysis used one Sentinel-2 scene and a common 3 × 3 mean filter, so it quantifies sensitivity to index-aggregation order rather than the effect of retraining on alternative LR–HR pairs. The cross-sensor test used Landsat-8 and Sentinel-2 acquisitions two days apart; its scores therefore characterize transfer for that paired acquisition and processing chain. Although the training archive covers geographically diverse regions, quantitative validation used the Anhui and Zhengzhou Sentinel-2 sets and one Landsat-8/Sentinel-2 pairing. The workflow can be applied to other study areas, while evaluation across additional regions, seasons, scales, and indices would extend the evidence. We compared established CNN architectures; a controlled comparison with attention- and Transformer-based models under the same single-band data and training protocol is a useful next step. The learned models retained their canonical objectives (MSE for SRCNN and VDSR; L1 for EDSR). Because we did not conduct a controlled loss ablation, we cannot separate the effects of architecture and objective; future work could use a common objective to support an architecture-focused comparison.

## 5. Conclusions

This study presents a two-part evaluation of single-band NDVI spatial downscaling: a controlled synthetic Sentinel-2 benchmark and a separate held-out Landsat-8/Sentinel-2 transfer assessment. We evaluated the published EDSR architecture without architectural modification and compared it with bicubic interpolation, SRCNN, and VDSR.

(1) On the Anhui validation set (330 patches) and Zhengzhou test set (300 patches), all three learned CNNs outperformed bicubic interpolation, with a consistent ranking of EDSR > VDSR > SRCNN. EDSR achieved 35.92 dB PSNR and 0.924 SSIM on validation, and 35.70 dB PSNR and 0.920 SSIM on test. Its paired mean PSNR gain over bicubic on the test set was 2.03 dB (95% bootstrap CI, 1.97–2.10 dB), indicating improved reconstruction under the defined synthetic 3× NDVI protocol.
(2) On the 300-patch Zhengzhou test set, EDSR had the lowest Sobel-gradient-magnitude MAE (0.01387) among the methods. This result complements the patch-wise reconstruction metrics with a comparison of local gradient magnitude.
(3) On the 272 held-out Landsat-8/Sentinel-2 pairs, EDSR achieved a mean PSNR of 25.58 dB, SSIM of 0.728, and R^2^ of 0.776 before shared linear calibration, and 26.40 dB, 0.770, and 0.810 after calibration. EDSR ranked first among the four tested methods and retained a 0.62-dB mean PSNR gain over bicubic after calibration. Its paired mean PSNR difference from VDSR was 0.232 dB (95% bootstrap CI, 0.203–0.263).
(4) The EDSR advantage was larger under controlled synthetic testing than in the cross-sensor evaluation. Reporting both results distinguishes reconstruction under a fixed degradation operator from transfer across the available satellite acquisitions.

Future work should test blind and kernel-aware degradation models, fine-tune models on real paired observations, and compare recent attention- and Transformer-based methods such as RCAN and SwinIR under the same single-band protocol. Evaluation across additional scale factors, vegetation indices, regions, and biomes would further test the transferability of the findings.

## Figures and Tables

**Table 1:** Datasets used in this study and their roles.

Dataset	Pairs	Sensor/Source	Scene ID and Acquisition Date
Training	2200	Sentinel-2 MSI, public dataset [20]	/
Validation	330	Sentinel-2 MSI L2A, Anhui	T50RPU_20210918T024551
Test	300	Sentinel-2 MSI L2A, Zhengzhou	T49SGU_20230716T030531
Cross-sensor pair pool	389	Landsat-8 OLI L2 + Sentinel-2 MSI L2A, Zhengzhou	LC08_L2SP_124036_20230515_20230523_02_T1 S2A_MSIL2A_20230517T030521_N0509_R075_T49SGU_20230517T080949.SAFE

**Table 2:** Architecture and training configuration of the compared models.

Item	SRCNN	VDSR	EDSR
Network depth	3 convolutional layers	20 layers	64 residual blocks
Feature channels	64/32	64	128
Main kernel sizes	9 × 9, 1 × 1, 5 × 5	3 × 3	3 × 3
Upsampling	Bicubic interpolation	Bicubic interpolation	PixelShuffle × 3
Residual scaling	/	/	0.1
Parameters (M)	0.008	0.666	20.369
Loss function	MSE loss	MSE loss	L1 loss

**Table 3:** Experimental environment and training settings.

Item	Setting
Operating system	Windows 10 Pro (64-bit)
CPU	Intel Xeon W-2155 @ 3.30 GHz
GPU	NVIDIA Quadro P4000
Framework	PyTorch 2.5.1 (CUDA 12.4), Python 3.10.20
Key libraries	NumPy 2.2.6, Rasterio 1.4.4, scikit-image 0.25.2
Optimizer	Adam
Learning rate	1 × 10^−4^
Batch size	SRCNN: 16; VDSR: 2; EDSR: 2
Maximum epochs	100
Full tile size (LR/HR)	170 × 170/510 × 510 pixels
Training crop size (LR/HR)	SRCNN: 96 × 96/288 × 288; VDSR and EDSR: 48 × 48/144 × 144

**Table 4:** Performance on the Anhui validation set (330 pairs). The best values are in bold.

Method	PSNR (dB)	SSIM	MAE	RMSE	R^2^
Bicubic	33.84	0.882	0.0266	0.0421	0.903
SRCNN	34.47	0.901	0.0251	0.0391	0.916
VDSR	35.63	0.918	0.0219	0.0344	0.935
**EDSR**	**35.92**	**0.924**	**0.0211**	**0.0333**	**0.938**

**Table 5:** Performance on the Zhengzhou test set (300 pairs). The best values are in bold.

Method	PSNR (dB)	SSIM	MAE	RMSE	R^2^
Bicubic	33.67	0.877	0.0275	0.0428	0.895
SRCNN	34.30	0.896	0.0260	0.0398	0.909
VDSR	35.42	0.914	0.0227	0.0352	0.929
**EDSR**	**35.70**	**0.920**	**0.0219**	**0.0340**	**0.933**

**Table 6:** Uncorrected cross-sensor performance on Landsat-8/Sentinel-2 evaluation pairs.

Method	PSNR (dB) ↑	SSIM ↑	RMSE ↓	MAE ↓	R^2^ ↑
Bicubic	24.9709	0.6639	0.1149	0.0894	0.7439
SRCNN	25.2881	0.7067	0.1109	0.0855	0.7615
VDSR	25.3215	0.7149	0.1106	0.0850	0.7624
EDSR	25.5840	0.7279	0.1073	0.0828	0.7764

**Table 7:** Bias-corrected cross-sensor performance on Landsat-8/Sentinel-2 evaluation pairs.

Method	PSNR (dB) ↑	SSIM ↑	RMSE ↓	MAE ↓	R^2^ ↑
Bicubic	25.7727	0.7107	0.1050	0.0784	0.7833
SRCNN	26.0710	0.7500	0.1016	0.0749	0.7963
VDSR	26.1649	0.7603	0.1007	0.0739	0.7997
EDSR	26.3973	0.7703	0.0979	0.0723	0.8102

Note: The arrows indicate changes relative to the baseline group results.

## Data Availability

Sentinel-2 Level-2A and Landsat-8 Level-2 imagery data are openly available from the respective agency data services.
